# Soymilk intake has desirable effects on phosphorus and calcium metabolism

**DOI:** 10.3164/jcbn.17-79

**Published:** 2018-02-17

**Authors:** Masae Sakuma, Ayaka Suzuki, Minako Kikuchi, Hidekazu Arai

**Affiliations:** 1 Laboratory of Clinical Nutrition and Management, Graduate School of Nutritional and Environmental Sciences, University of Shizuoka, 52-1 Yada, Suruga-ku, Shizuoka 422-8526, Japan; 2 Department of Human Nutrition, School of Life Studies, Sugiyama Jogakuen University, 17-3 Hoshigaoka Motomachi, Chikusa-ku, Nagoya 464-8662, Japan

**Keywords:** soymilk, dietary phosphorus, serum phosphorus level, parathyroid hormone

## Abstract

The objective was to evaluate the effect of replacing milk with soymilk or calcium-fortified soymilk as a part of a meal on postprandial serum phosphorus levels. This study had a randomized crossover design. Ten healthy subjects were enrolled and consumed three test meals that contained either milk, soymilk, or calcium-fortified soymilk containing the same amount of calcium as milk. Blood samples were collected at 0, 30, 60, 120, 240 and 360 min and urine samples were collected from 0 to 360 min after consuming the test meal. Serum phosphorus levels decreased the most after the ingestion of the soymilk meal, and the least after the ingestion of the milk meal. After the ingestion of each meal, serum intact parathyroid hormone levels showed an initial drop followed by a gradual rise, and these changes were more pronounced for the soymilk meal than for the milk meal and the soymilk + calcium meal. Our study shows that replacing milk with soymilk as a part of a meal may suppress the postprandial elevation in serum phosphorus levels, even when the soymilk contains the same amount of calcium as milk.

## Introduction

In many countries, dietary phosphorus intake is increasing. It was reported that phosphorus intake in western countries is two- to three-fold higher than the nutritional recommendations due to an abundant consumption of dairy and meat products.^(1)^ In Japan, the consumption of plant products such as rice has decreased, while the consumption of animal products such as meat and dairy has increased, because a westernized diet has become more popular among Japanese people (http://www.e-stat.go.jp/SG1/estat/List.do?lid=000001131797). Phosphorus in animal products has a higher absorption rate following ingestion than that in plant products.^(2)^ Elevated serum phosphorus levels are known to promote vascular calcification, arterial sclerosis, and cardiovascular diseases,^(3–5)^ and have been associated with mortality in dialysis patients.^(6,7)^ Evidence suggests that hyperphosphatemia may induce cardiovascular events, even in individuals with normal renal function.^(8–10)^ Therefore, it is recommended that a high dietary intake of phosphorus should be avoided to maintain serum phosphorus levels within the appropriate range.

Conversely, calcium intake remains below nutritional recommendations.^(11–13)^ In an animal study, a diet with a low calcium: phosphorus ratio triggered secondary hyperparathyroidism, loss of bone, and osteopenia.^(14)^ Another study suggested that the habitual intake of a diet with a low calcium: phosphorus ratio may interfere with the homoeostasis of calcium metabolism and cause increased bone resorption in healthy women.^(15)^ Furthermore, the intake of a diet with a high calcium:phosphorus ratio due to a low phosphorus content was effective for promoting bone mineralization in adult female rats due to an increase in calcium absorption.^(16)^ The consumption of >1,000 mg/day calcium and a dietary calcium: phosphorus ratio of >0.74 were associated with better bone mineral density values in young women.^(17)^ Therefore, increasing the dietary calcium: phosphorus ratio by maintaining a low phosphorus intake and a high calcium intake is desirable for vascular and bone health.

Parathyroid hormone (PTH) is a regulator of phosphate homeostasis. Serum phosphorus levels are homeostatically maintained through a complex interplay between intestinal absorption and renal excretion. PTH secretion is promoted by a rise in the serum phosphorus level, and it acts to increase urinary phosphorus excretion by decreasing renal phosphate reabsorption via lowering the expression of renal sodium-phosphate transporter (NaPi) 2a and NaPi 2c.^(18–20)^ PTH regulates not only phosphorus metabolism but also calcium metabolism. PTH stimulates calcium release from bone and enhances the intestinal absorption of calcium by elevating the renal production of calcitriol 1,25(OH)_2_D_3_.^(21)^ The secretion of PTH is induced by lowering serum calcium levels^(22)^ and diminished by increasing serum calcium levels.^(23)^

Soymilk and milk are consumed in similar situations. Soymilk is a plant product, so the intestinal absorption rate of phosphorus from soymilk is low. By contrast, milk, which is an animal product, provides a high intestinal absorption rate of phosphorus. In addition, milk is rich in calcium, so urinary phosphorus excretion is decreased after milk ingestion due to a lower secretion of PTH. Consequently, it has been suggested that milk ingestion can easily lead to hyperphosphatemia. In a previous study, serum phosphorus levels were higher in individuals who ingested dairy products than in individuals who ingested other animal and plant products.^(24)^ Although replacing milk with soymilk may effectively control serum phosphorus, the low calcium content of soymilk is deleterious to bone health. However, whether the suppressive effect of soymilk on serum phosphorus levels is maintained even when the soymilk is fortified with the same amount of calcium contained in milk has not been clarified. Thus, the objective of this research was to determine the effect of replacing milk with soymilk as a part of a meal on serum phosphorus levels even when the soymilk contains the same amount of calcium as milk.

## Materials and Methods

### Subjects and protocol

The study was performed after obtaining written informed consent from all subjects, and the protocol was approved by the Ethics Committee of the University of Shizuoka. The protocol conformed to the Helsinki Declaration.

For this experiment, 10 healthy subjects without pre-existing conditions or medication use were enrolled. The mean values ± SD for age and body mass index were 22.4 ± 1.2 years and 20.3 ± 6.3 kg/m^2^, respectively. The characteristics of the subjects are shown in Table 1.

This study had a randomized crossover design. The experiment was conducted so that each test day was separated by a washout period of 7 days. On the day before each test day, subjects were requested to refrain from heavy exercise and alcohol consumption, and to fast from 15:00 onwards. A pre-specified meal was provided by 20:00. After an overnight fast, subjects visited the laboratory at 08:15 and were asked to void. Fasting venous blood samples were collected at 08:30. Subjects then consumed the test meal with a known phosphorus content at 08:45 and were required to consume each test meal within 15 min. Blood samples were collected at 0 min (i.e., immediately before the meal) and at 30, 60, 120, 240 and 360 min after the meal. All subjects drank the appointed phosphorus-free water at a rate of 100 ml/h during the experimental period. During the experimental period, subjects were asked to abstain from foods and beverages other than test meals and the appointed phosphorus-free water. Urine samples were collected from 0 to 360 min after consuming the test meal.

### Clinical trials

This trial was not registered in publicly accessible database because this study conducted as preliminary trial.

### Test meals

The nutrient compositions of the test meals are shown in Table 2. The effects of milk and soymilk were compared between three test meals consisting of 120 g wheat bread, 14 g strawberry jam, 8 g margarine, and either 225 g milk (Morinaga Milk Industry Co., Tokyo, Japan) (Milk-meal), 335 g unadjusted soymilk (Marusan-Ai Co., Aichi, Japan) (Soymilk-meal), or 200 g calcium-fortified soymilk (Otsuka Chilled Foods Co., Tokyo, Japan) + 105 g unadjusted soymilk (Soymilk + Ca-meal). To equalize the volumes of the three test meals, water was added to the Milk-meal and the Soymilk + Ca-meal.

### Anthropometric and blood analysis

Height, body weight, and body fat percentage were calculated using a bioelectrical impedance analysis method (Tanita TBF-215; Tanita Corporation, Tokyo, Japan). Blood samples were dispensed into vacuum tubes and immediately centrifuged (4°C, 1,500 × *g*, 10 min). Serum and plasma were separated, and the samples were stored at −30°C until the analysis of serum phosphorus (S-Pi), serum calcium (S-Ca), serum intact parathyroid hormone (S-PTH), serum insulin (S-IRI) and plasma glucose (P-G) levels by a commercial laboratory (SRL, Inc., Tokyo, Japan).

### Urine collection method

Urine samples were collected from 0 to 360 min after consuming the test meal. After recording the total volume, urine samples were dispensed into vessels for storage at 4°C until analysis. Urine inorganic phosphorus (U-Pi) and urine calcium (U-Ca) levels were measured by a commercial laboratory (SRL, Inc.).

### Statistical analysis

Data are expressed as the means ± SD. *P* values <0.05 were considered to denote statistical significance. The Shapiro-Wilk test was used to determine normality. The significance of differences in serum and plasma parameters among the diet groups was calculated using a paired two-way analysis of variance with Tukey’s post-hoc test. Differences in urine parameters were calculated using a one-way ANOVA with Tukey’s post-hoc test. All statistical analyses were performed using SPSS ver. 22.0 software (SPSS Inc., Chicago, IL).

## Results

### Serum phosphorus, calcium and intact PTH levels

**Fig. 1:**
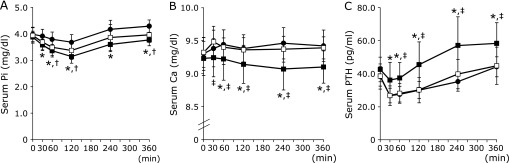
Changes in serum phosphorus, calcium and intact parathyroid hormone levels. (A) serum phosphorus levels, (B) serum calcium levels, (C) serum intact parathyroid hormone levels. Closed circle; Milk-meal, closed square; Soymilk-meal, open square; Soymilk + Ca-meal. Values are means ± SD. *Denotes significant differences between the Milk-meal and Soymilk-meal groups (*p*<0.05). ^†^Denotes significant differences between the Milk-meal and Soymilk + Ca-meal groups (*p*<0.05). ^‡^Denotes significant differences between the Soymilk-meal and Soymilk + Ca-meal groups (*p*<0.05).

 The variations in the S-Pi, S-Ca and S-PTH levels are shown in Fig. 1. S-Pi decreased after all meals from 0 to 120 min. The largest decrement of S-Pi occurred after the ingestion of the Soymilk-meal, and the smallest occurred after the ingestion of the Milk-meal. The S-Pi levels at 30, 60 and 120 min for the Soymilk-meal were significantly lower than those for the Milk-meal (*p*<0.05), and the S-Pi levels at 60 and 120 min for the Soymilk + Ca-meal were significantly lower than those for the Milk-meal (*p*<0.05). After the 120-min time point, S-Pi gradually increased and returned to fasting levels at 360 min for the Soymilk-meal and the Soymilk + Ca-meal, but S-Pi at 360 min for the Milk-meal was above the fasting level (Fig. 1A).

Postprandial S-Ca showed an increasing trend from the fasting level after the ingestion of the Milk-meal and the Soymilk + Ca-meal. By contrast, postprandial S-Ca showed a decreasing tendency from the fasting level after the intake of the Soymilk-meal. S-Ca for the Soymilk-meal was markedly lower than that for the Milk-meal at 60, 120, 240 and 360 min (*p*<0.05), and lower than that for the Soymilk + Ca-meal at 30, 60, 120, 240 and 360 min (*p*<0.05) (Fig. 1B).

S-PTH decreased after the ingestion of all test meals at 30 min, and then increased over time. The changes of S-PTH for the Milk-meal and the Soymilk + Ca-meal, which had the same calcium content, were highly similar. Postprandial S-PTH after the ingestion of the Soymilk-meal was significantly higher than that for the Milk-meal and the Soymilk + Ca-meal at 30, 60, 120, 240 and 360 min (*p*<0.05) (Fig. 1C).

### Urine phosphorus and calcium

**Fig. 2:**
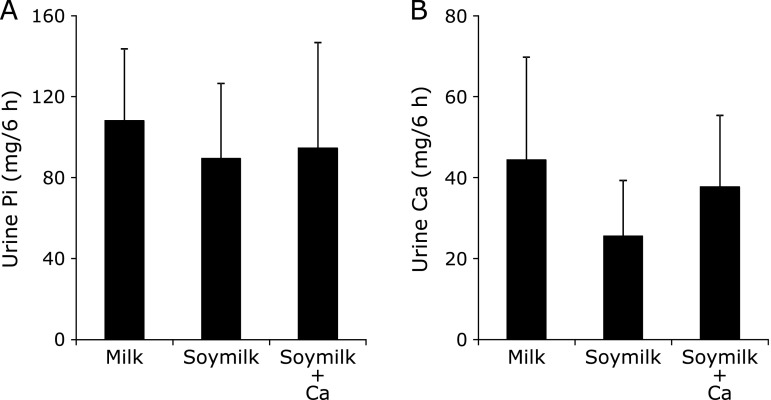
Averages in urine phosphorus and calcium excretions. (A) urine phosphorus excretions, (B) urine calcium excretions. Values are means ± SD.

Urine phosphorus (U-Pi) and calcium (U-Ca) did not differ significantly among the meal groups (Fig. 2).

### Serum insulin and plasma glucose levels

S-IRI increased after each meal and peaked at 30 min, then gradually returned to the fasting level. There were no significant differences in S-IRI responses among meal groups (data not shown). P-G after the ingestion of each test meal increased and peaked at 30 min. P-G responses did not differ significantly among the meal groups (data not shown).

## Discussion

In this study, we examined the effects of soymilk on the control of serum phosphorus levels. S-Pi for the Soymilk-meal remained at a lower level than that for the other two test meals, and S-Pi for the Soymilk + Ca-meal remained at a lower level than that for the Milk-meal during the experimental period. The elevation of S-Pi for the Milk-meal was small and did not induce hyperphosphatemia. However, previous studies reported that S-Pi levels are lowest in the morning, rise over time, and are highest in the middle of the night.^(25,26)^ So S-Pi levels may have increased further when the Milk-meal was consumed at lunch or dinner. There are two major possible reasons for the observed results for S-Pi.

First, the effect of a difference in the absorption rate of phosphorus was considered. Phosphorus is classified as inorganic phosphorus, which is included in food additives, or organic phosphorus, which is contained in natural foods. Inorganic phosphorus has a higher absorption rate than organic phosphorus because it can be easily released in the intestines.^(24)^ Organic phosphorus can be grouped into animal-derived and plant-derived phosphorus. Animal-derived phosphorus is hydrolyzed in the intestines and then absorbed into the circulation as inorganic phosphorus.^(27)^ Additionally, plant-derived phosphorus mostly exists in the form of phytic acid or phytate.^(28,29)^ Humans do not express the phytate-degrading enzyme phytase, so the bioavailability of plant-derived phosphorus is lower than that of animal-derived phosphorus. Since soymilk is a plant product, the absorption rate of the phosphorus contained in soymilk is lower than that of the phosphorus contained in milk, which is an animal product. Such a difference in absorption rate may have contributed to the significantly lower S-Pi for the Soymilk-meal than for the Milk-meal.

The second possible factor affecting serum phosphorus levels is the effect of PTH. PTH increases the urinary excretion of phosphorus by lowering renal sodium-phosphate transporter (NaPi-2a and NaPi-2c) expression.^(20)^ In addition, the synthesis and secretion of PTH are strictly regulated by serum calcium levels, which are sensed by the calcium-sensing receptor (CaSR). An elevated extracellular concentration of calcium activates CaSR and inhibits PTH synthesis and release into the blood.^(30)^ In this study, the S-Ca levels for the Milk-meal and the Soymilk + Ca-meal were markedly higher than that for the Soymilk-meal. Furthermore, postprandial S-PTH levels after the ingestion of the Milk-meal and the Soymilk + Ca-meal were significantly lower than that for the Soymilk-meal. The postprandial changes in S-PTH for the Milk-meal and the Soymilk + Ca-meal, which have the same calcium content, were highly similar. In fact, PTH was secreted according to the amount of calcium in the test meal. Because urinary phosphorus excretion reflects phosphorus absorption in the intestine,^(31)^ it was calculated that U-Pi for the Milk-meal was higher than that for the other two meals, but U-Pi did not differ significantly among the meal groups. The following can be considered as the cause of these results. Since the Milk-meal and the Soymilk + Ca-meal were rich in calcium, the enhancement of PTH secretion could not have been caused by increased S-Ca levels. Thus, urinary phosphorus excretion was suppressed after the ingestion of the Milk-meal and the Soymilk + Ca-meal. The reason for the lack of difference in U-Pi among the meal groups appears to be due to an interaction between phosphorus absorption and urinary phosphorus excretion. High S-Pi levels promote vascular calcification and arterial sclerosis, so it can be considered that the replacement of milk with soymilk in the diet is effective for maintaining vascular health.

Additionally, a low dietary calcium: phosphorus ratio was reported to triggered secondary hyperparathyroidism with bone loss and osteopenia^(14)^ or bone resorption.^(15)^ Therefore, increasing the dietary calcium: phosphorus ratio by lowering phosphorus intake and increasing calcium intake is desirable for bone health. In this study, although S-Ca, S-PTH, and U-Pi did not differ between the Soymilk + Ca-meal and Milk-meal groups, S-Pi for the Soymilk + Ca-meal was lower than that for the Milk-meal. These results may derive from the difference in phosphorus absorptivity between milk and soymilk. The Soymilk + Ca-meal avoids the disadvantage of soymilk, which is that its calcium content is low compared with that of milk. Additionally, the Soymilk + Ca-meal maintained the advantage of soymilk, which is that it suppresses the postprandial rise in S-Pi.

An increase in plasma glucose evokes insulin release, causing an increased cellular uptake of phosphorus along with glucose.^(32)^ In this study, it was thought that the influences of S-IRI and P-G on S-Pi were almost equal for all test meals because there were no significant differences in the postprandial responses of S-IRI and P-G among the meal groups.

Our study shows that replacing milk with soymilk as a part of a meal may suppress the elevation in serum phosphorus levels. It is important to ensure that the effect of soymilk, which suppresses the postprandial elevation of serum phosphorus levels, was maintained even when the soymilk was fortified to contain the same amount of calcium as milk. In summary, our data suggest that calcium-fortified soymilk may be effective for maintaining vascular and bone health. Because sample size of this study is small, further study is needed to confirm these finding.

## Figures and Tables

**Table 1:** Characteristics of the subjects

Characteristic	Mean ± SD
Subjects (M/F)	(4/6)
Age (year)	22.4 ± 1.2
Height (cm)	164.4 ± 6.5
Body weight (kg)	54.9 ± 6.0
BFP (%)	21.2 ± 1.7
BMI (kg/m^2^)	20.3 ± 6.3
Pi (mg/dl)	3.9 ± 0.3
Ca (mg/dl)	9.3 ± 0.2
intact PTH (pg/dl)	40 ± 7.1
FPG (mg/dl)	89 ± 4.0
IRI (µU/ml)	4.6 ± 1.2

Values are mean ± SD. BFP, body fat percentage; BMI, body mass index; Pi, phosphorus; Ca, calcium; intact PTH, intact parathyroid hormone; FPG, fasting plasma glucose; IRI, immunoreactive insulin.

**Table 2:** Composition of the test meals

Intervention and meal	Serving size (g)	Energy (kcal)	Protein (g)	Fat (g)	Carbohydrate (g)	Phosphorus (mg)	Calcium (mg)	Sodium (mg)
Milk-meal								
Milk	225	150	7.4	8.9	11	200	252	97.0
Water	110	0	0	0	0	0	0	0
Wheat bread	120	317	11.2	5.3	56	100	35	1.6
Strawberry jam	14	36	0.1	0	9	2	1	0
Soft margarine	8	62	0	6.6	0	1	1	0.1
**Total**		565	18.7	20.8	76	303	289	98.7
								
Soymilk-meal								
Unadjusted soymilk	335	174	15.7	10.4	4	200	42	3.4
Wheat bread	120	317	11.2	5.3	56	100	35	1.6
Strawberry jam	14	36	0.1	0	9	2	1	0
Soft margarine	8	62	0	6.6	0	1	1	0.1
**Total**		589	27.0	22.3	69	303	79	5.1
								
Soymilk + Ca-meal								
Ca fortified soy milk	200	95	5.4	4.5	9	137	234	0
Unadjusted soymilk	105	55	4.9	3.3	1	63	13	1.1
Water	30	0	0	0	0	0	0	0
Wheat bread	120	317	11.2	5.3	56	100	35	1.6
Strawberry jam	14	36	0.1	0	9	2	1	0
Soft margarine	8	62	0	6.6	0	1	1	0.1
**Total**		565	21.6	19.7	75	303	284	2.8
